# Upper eyelid subcutaneous orbital fat prolapse: A case series on a condition that deserves a separate name

**DOI:** 10.1016/j.heliyon.2024.e37982

**Published:** 2024-09-16

**Authors:** Bin Hu, Yun Liu, Yingli Ji, Chen Zhang, Yanming Tian

**Affiliations:** a School of Medicine, Shihezi University, Shihezi, China; b Department of Gynecology and Obstetrics, Xinjiang, 474 Hospital, Urumqi, China; c Department of Ophthalmology, Xinjiang, 474 Hospital, Urumqi, China; d Clinical College of Ophthalmology, Xinjiang Medical University, Urumqi, China; e Department of Ophthalmology, General Hospital of Xinjiang Military Region of the Chinese People's Liberation Army, Urumqi, China

**Keywords:** Periorbital fat, The upper eyelid, Differential diagnosis

## Abstract

In the field of ophthalmology, orbital fat prolapse under the upper eyelid is less recognized than subconjunctival orbital fat prolapse. Despite its occasional occurrence in clinical practice, this condition is often inadequately understood and incorrectly classified due to its subtle manifestations and the limited number of focused studies. Typically affecting young patients, the prolapse is located subcutaneously in the mid-upper eyelid, resulting in a pseudo-occlusion. This study aims to investigate this specific ocular anomaly, we delineate the clinical presentations, surgical interventions, and differential diagnoses of this condition through three representative cases, proposing its classification as a distinct disease entity named Upper Eyelid Subcutaneous Orbital Fat Prolapse (UESOFP).

## 1 Introduction

In clinical practice, orbital fat prolapse primarily refers to subconjunctival orbital fat prolapse. However, some cases deviate from this typical presentation, necessitating a thorough differential diagnosis. These cases should be classified as Upper Eyelid Subcutaneous Orbital Fat Prolapse (UESOFP), distinct from the subconjunctival type. To assist ophthalmologists with precise diagnosis, we offer a comprehensive description of the clinical features, illustrated with examples from representative cases.

## 2 Case presentation

**Fig. 1:**
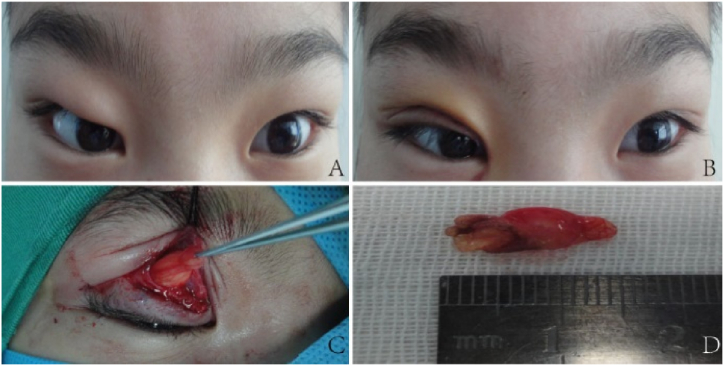
A: Swollen right upper eyelid, absent double eyelid, and elevated right eyebrow. B: Post-surgical recovery of double eyelid, with reduced eyebrow elevation. C: Prolapsed orbital fat exposed through double eyelid incision. D: Removed orbital fat.

Case 1: A 12-year-old Han Chinese girl with a >2-year history of unexplained right upper eyelid swelling was admitted to the hospital on March 4, 2015 (Fig. 1A). She was otherwise healthy, with no history of trauma or hereditary eye diseases in her family. There was no record of previous ocular surgery or trauma. The patient experienced significant swelling and discomfort in the right upper eyelid; however, no prolapse was observed under the conjunctiva during the examination. A right orbital lipectomy was subsequently performed on March 6, 2015. Upon surgical exploration, it was determined that the upper eyelid protrusion was due to prolapse of orbital fat into the subcutaneous tissue (Fig. 1C and D). Postoperatively, her double eyelid restored, and her eyebrow elevation was reduced, with no significant complications (Fig. 1B).

**Fig. 2:**
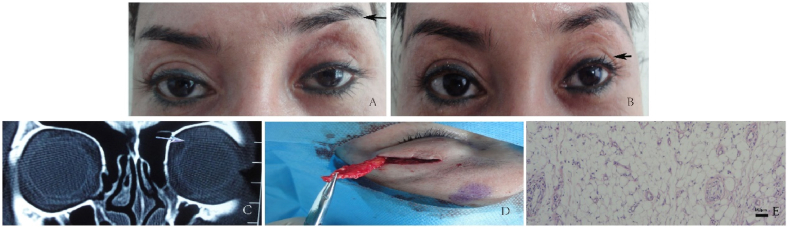
A: Disappearance of the left double eyelid, slight ptosis related to pressure on the upper eyelid, and elevated left eyebrow (black arrow). B: One month after surgery, slight relief of eyebrow elevation, and partial restoration of the double eyelid (black arrow). C: A medium-to low-density lesion with unclear boundaries in the anterior upper orbital septum of the left eye (white arrow). D: Sub-brow incision with excised prolapsed fat and partial fibrous proliferation. E: Pathological examination.

Case 2: A 41-year-old Uyghur woman with a four-month history of unexplained heaviness in the left upper eyelid was admitted to the hospital on August 1, 2014. Her left upper eyelid had lost its double eyelid fold, and the increased upper eyelid pressure caused slight ptosis, with elevation of the left eyebrow (Fig. 2A). Notably, she had previously undergone a forehead hyaluronic acid filler injection in November 2010 to address facial skin laxity. CT imaging revealed a medium-to low-density lesion with unclear boundaries in the anterior upper orbital septum of the left eye (Fig. 2C). On August 6, 2014, a sub-brow incision was performed, through which the prolapsed fat and areas of partial fibrous proliferation were excised (Fig. 2D); some lobules of the lacrimal gland were present in the excised tissue. Pathological examination revealed a significant increase in adipocytes, accompanied by fibroplasia and lymphocytic infiltration (Fig. 2E). One month after the surgery she experienced improvements, including alleviation of left eyelid ptosis, a slight reduction in eyebrow elevation, and partial restoration of the double eyelid fold (Fig. 2B).

**Fig. 3:**
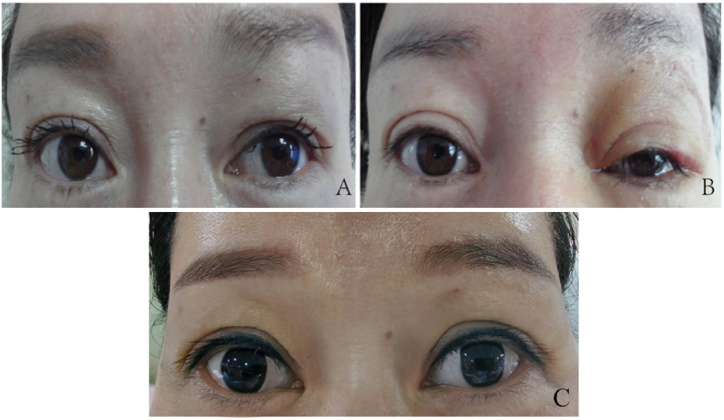
A: Fullness in the middle of the left upper eyelid, along with left eyebrow elevation above the right eyelid, and asymmetric double eyelids. B: Four days after surgery for excised prolapsed fat via sub-brow incision; results include mild swelling in the left upper eyelid and partial restoration of the left double eyelid. C: Nine years after surgery, the left double eyelids were fully recovered and both eyes were symmetrical in appearance.

Case 3: A 38-year-old Han Chinese woman with a history of unexplained swelling in the left upper eyelid for over a year visited the hospital on March 4, 2015. She presented with fullness in the middle of the upper eyelid, elevation of the left eyebrow above the right, and noticeable asymmetry in her double eyelids (Fig. 3A). She had no history of hypertension, diabetes, heart disease, or other chronic illnesses and had not received any prior treatment or consultations for the eyelid swelling. On March 10, 2015, we dissected and excised the prolapsed orbital fat from the incision site on the upper eyelid for her. The postoperative recovery was smooth, with no significant complications. Four days postoperatively, she exhibited mild swelling in the left upper eyelid and partial recovery of the left double eyelid (Fig. 3B). Nine years later, the patient's eyelid edema had completely resolved, with full restoration of the double eyelid and symmetrical appearance of both eyes (Fig. 3C).

## 3 Discussion

UESOFP typically occurs at a younger age, unlike subconjunctival orbital fat prolapse, which predominantly affects older men [1]. This condition is more common in children or young women, and it lacks distinct causative factors. Initially, individuals may notice eyelid swelling or fullness; upon examination, a soft, subcutaneous mass with a relatively fixed base is revealed. The subcutaneous mass can be partially repositioned upon compression but re-emerges when the pressure is released. Subcutaneous fat prolapse may alter the upper eyelid creases, leading to asymmetry between the eyelids or disappearance of the crease in the affected eye. To elevate the affected upper eyelid, patients often exaggerate the use of their forehead muscles, resulting in a higher eyebrow than on the healthy side. As the condition progresses, a prominent bulge might develop in the upper eyelid skin, considerably altering its appearance. Orbital computed tomography (CT) scans may reveal the presence of clustered medium-to low-density lesions in the anterior region of the orbital septum in the affected eye, with unclear boundaries. These medium-density lesions are associated with the proliferation of fibrous connective tissue, stimulated by the fat prolapse.

Subcutaneous upper eyelid orbital fat prolapse often affects appearance and typically requires surgical intervention. Depending on the location of the prolapsed orbital fat, the surgery involves either a sub-brow incision or a double eyelid incision. Once the skin and subcutaneous layer are incised, the prolapsed fat and defective orbital septum are exposed. The prolapsed fat originating from the normal orbital fat behind the superior orbital septum, is then clamped at the base with parallel orbital septum forceps, electrically coagulated, and excised. The local orbital septum is then sutured followed by suturing of the subcutaneous layer and skin. The excision is limited to the prolapsed orbital septal fat pad, avoiding excessive traction to prevent over-removal of intraorbital fat, which could result in localized concavity. During the surgery, there is no interference with the levator palpebrae muscle. Given the elasticity and adaptability of the skin and tissues in the eyelid region, we believe that the eyelids can naturally adjust and recover postoperatively, even after the removal of a significant amount of prolapsed fat. Therefore, we do not perform additional postoperative decompression. The sub-brow incision has minimal impact on appearance, and as the wound heals, the double eyelid gradually becomes more defined. Although our findings indicate promising outcomes, the small sample size and brief follow-up period impose limitations that may compromise the generalizability of the results. Future studies should address these limitations by including larger, multi-center cohorts with extended follow-up durations to better validate and generalize the findings.

Orbital fat prolapse is common in older adults, and its pathogenesis is related to age-related weakening of the orbital septum. In cosmetology, orbital fat prolapse in the lower eyelid is often referred to as “eyelid bags.” Unspecified references to orbital fat prolapse typically imply subconjunctival orbital fat prolapse. This condition is uncommon; most instances have been documented via case reports [[2], [3], [4]]. For example, a 79-year-old man presented with subconjunctival orbital fat prolapse, accompanied by subcutaneous orbital fat prolapse in the upper and lower eyelids [2]. The authors termed this condition “extraconal fat prolapse,” based on orbital imaging segmentation work by Muller-Forell. However, this terminology does not specify the precise location of prolapse within the orbit. The cases described in the present study considerably differ from the well-known subconjunctival orbital fat prolapse. First, UESOFP tends to occur in younger individuals, suggesting a pathogenesis distinct from the age-related weakening that typically causes orbital fat prolapse in older adults. Second, the site of UESOFP varies, whereas subconjunctival prolapse usually occurs beneath the conjunctiva in the superior temporal region. Some researchers have noted the presence of a third fat pad in the upper eyelid during blepharoplasty, which protrudes from the lower edge of the lacrimal gland and causes fullness or bulging in the lateral third of the upper eyelid in some patients [5]. In contrast, the cases in the present study exhibited a central bulge in the upper eyelid, accompanied by compensatory eyebrow elevation. Extensive investigations are required to elucidate the specific underlying pathogenesis. Some patients with chronic lacrimal gland prolapse exhibit clinical features similar to UESOFP. However, lacrimal gland prolapse is characterized by a palpable, firm, and well-defined movable mass in the lacrimal region of the upper eyelid, which can be repositioned with pressure and is identifiable on CT as a prolapsed lacrimal gland. Conversely, UESOFP is typically located in the central part of the eyelid, with softer, indistinct fat that manifests as unclear lesions without lacrimal gland features on CT. Furthermore, it is crucial to differentiate UESOFP from blepharitis, which is commonly defined by eyelid swelling, inflammation (redness and thickening) of the lid margins, discomfort, potential discharge, and drooping of the upper eyelids. Blepharitis is typically attributed to bacterial infection or other inflammatory factors. In contrast, UESOFP manifests with edema and the formation of bags in the upper eyelid region, often without substantial inflammation of the eyelid skin. These characteristics support the independent classification of such manifestations as “Upper Eyelid Subcutaneous Orbital Fat Prolapse.” This distinction aims to improve clinical diagnosis, treatment, and differentiation. Further research is needed to elucidate the pathogenesis of this condition, distinct from subconjunctival orbital fat prolapse, which will help to refine treatment and prevention approaches.

## Data availability statement

Data will be made available on request.

## Ethical statement

Informed consent was obtained from all participants/patients (or their proxies/legal guardians) for the publication of all their data and/or images.

## CRediT authorship contribution statement

**Bin Hu:** Writing – original draft. **Yun Liu:** Investigation. **Yingli Ji:** Supervision. **Chen Zhang:** Data curation. **Yanming Tian:** Writing – review & editing, Conceptualization.

## Declaration of competing interest

The authors declare that they have no known competing financial interests or personal relationships that could have appeared to influence the work reported in this paper.
